# Editorial Note

**Published:** 1936

**Authors:** 


### Editorial Note

**British National Human Heredity Council.**

THE British National Human Heredity Committee was formed in 1932 for the collection of data and the investigation of human pedigrees, as a branch of the International Federation of Eugenic Organizations. It has now been enlarged as a Council, and in collaboration with the Galton Laboratory aims at setting up a Clearing House for material of Human Genetics.

The Council will be grateful to receive from institutions and individuals all available material which can furnish well authenticated data on the transmission of abnormal human traits of every kind. Family histories or pedigrees, twin studies and statistical researches are mainly contemplated. Any contributors of material who may wish to retain the sole right of publication or copyright are asked to accompany their contributions with a statement to that effect.

Reprints of published work will also be most acceptable. Some authors, when publishing material may also have collected a number of pedigrees which they have been unable to reproduce in detail. It is the hope of the Council that such records, by being included in the Clearing House, should not be lost. Copies of the standard International Pedigree Symbols may be obtained from the Office of the Council.

Contributions should be accompanied by all available details in regard to source, diagnostic symptoms, and the name and address of the person or persons who vouch for accuracy. All such details will be regarded as strictly confidential.

The other objects contemplated in this enterprise, namely facilities for study, replies to enquiries, and information service, cannot be initiated for some time. Announcement of these activities will be made later.

There can be few medical men who do not from time to time come across instances of hereditary disorders and defects. It is, however, difficult to secure publication of the pedigrees. Authors and editors naturally feel that it is not worth while describing one more family when many are already recorded in the literature. If, however, the science of human heredity is to progress it is most necessary that as many pedigrees as possible should be available for study.

